# The Current and Future Use of Ridge Regression for Prediction in Quantitative Genetics

**DOI:** 10.1155/2015/143712

**Published:** 2015-07-26

**Authors:** Ronald de Vlaming, Patrick J. F. Groenen

**Affiliations:** ^1^Erasmus University Rotterdam Institute for Behavior and Biology, Department of Applied Economics, Erasmus School of Economics, Erasmus University Rotterdam, Postbus 1738, 3000 DR Rotterdam, Netherlands; ^2^Econometric Institute, Erasmus School of Economics, Erasmus University Rotterdam, Postbus 1738, 3000 DR Rotterdam, Netherlands

## Abstract

In recent years, there has been a considerable amount of research on the use of regularization methods for inference and prediction in quantitative genetics. Such research mostly focuses on selection of markers and shrinkage of their effects. In this review paper, the use of *ridge regression* for prediction in quantitative genetics using *single-nucleotide polymorphism* data is discussed. In particular, we consider (i) the theoretical foundations of ridge regression, (ii) its link to commonly used methods in animal breeding, (iii) the computational feasibility, and (iv) the scope for constructing prediction models with nonlinear effects (e.g., *dominance* and *epistasis*). Based on a simulation study we gauge the current and future potential of ridge regression for prediction of human traits using genome-wide SNP data. We conclude that, for outcomes with a relatively simple genetic architecture, given current sample sizes in most cohorts (i.e., *N* < 10,000) the predictive accuracy of ridge regression is slightly higher than the classical *genome-wide association study* approach of *repeated simple regression* (i.e., one regression per SNP). However, both capture only a small proportion of the heritability. Nevertheless, we find evidence that for large-scale initiatives, such as biobanks, sample sizes can be achieved where ridge regression compared to the classical approach improves predictive accuracy substantially.

## 1. Introduction

The advent of large-scale molecular genetic data has paved the way for using these data to help predict, diagnose, and treat complex human diseases [1]. In recent years, the use of such data for the prediction of polygenic diseases and traits has become increasingly popular (e.g., [2–4]). This venue has proved successful even for traits such as educational attainment and cognitive performance [5, 6]. The vast majority of research into the genetic architecture of human traits and diseases is exploratory and considers the effects of at least hundreds of thousands of* single-nucleotide polymorphisms* (SNPs) on the outcome of interest [7].

Predictions based on molecular genetic data are typically constructed as a weighted linear combination of the available SNPs. This yields a so-called* polygenic risk score* [3] (*polygenic score*,* genetic risk score*, and* genome-wide score* [8]).* Multiple regression* (*ordinary least squares*, OLS) is a natural technique for estimating the weights of the predictors (SNPs) in this context but cannot be applied here: in general, the number of samples (*N*) available is far lower than the number of SNPs (*P*); typically, *N* < 10,000 and *P* > 100,000. OLS would yield a perfect in-sample prediction without any predictive value out of sample and would not allow drawing inferences on the weights of the SNPs, as they are nonunique. A commonly accepted solution to this problem is to carry out a* genome-wide association study* (GWAS), where one regresses the outcome of interest on each SNP separately. In this paper, we call this the* repeated simple regression* (RSR) approach.

Polygenic scores are typically constructed as the weighted sum of the SNPs with weights resulting from a GWAS using RSR. We raise four points of critique regarding this method. The first problem with this approach is that, in contrast to multiple regression, there is no search for the best linear combination over all SNPs jointly for predicting the outcome. A second, related, problem is that highly correlated SNPs (i.e., SNPs in strong* linkage disequilibrium*) repeatedly contribute very similar information, thereby distorting the risk score. For example, consider a set of ten perfectly correlated SNPs. In the RSR, they receive exactly the same weight. As the polygenic risk score is a weighted linear sum of the SNPs with the weights coming from RSR, these perfectly correlated SNPs contribute a factor ten stronger to the risk score than a single SNP capturing all information from that region does. This factor ten does not depend on the predictive power of the information in that region. A third problem is that the polygenic risk score can theoretically be correlated with* confounding variables* (*confounders*,* control variables*, and* controls*). For instance, SNPs can be correlated with the population structure. Therefore, the polygenic risk—being a linear combination of SNPs—can be correlated with the confounders. Usually, confounders, such as age and gender, are included as regressors in order to control for spurious relations through these covariates. However, we find that often in empirical work researchers do not control properly for the confounders in at least one of the many steps that lead from phenotype and genotype data to evaluation of the out-of-sample predictive accuracy of the polygenic risk score. A fourth problem is that the RSR approach is not able to handle even two-way interactions between the SNPs, as it would lead to a number of weights to be estimated that is quadratic in the number of SNPs, which is clearly computationally infeasible.

In this paper, we review the use of ridge regression (RR) [9] to tackle the four problems discussed above. The purpose of this paper is threefold. First, we discuss how prediction using RR can address the aforementioned four points of critique pertaining to a typical polygenic score, that is, how RR can be used to search for the best linear combination of SNPs jointly, to address the multicollinearity of SNPs [10, 11], and to account for the presence of confounding variables and of nonlinear SNP effects (e.g., [12–17]). Second, we review relevant work on ridge regression both in and outside the field genetics. Third, we assess the merits of prediction using ridge regression in the new domain of biobanks. That is, we predict the expected accuracy of ridge regression in large scale initiatives with over a 100,000 observations.

An important property of RR is that it cannot select a subset of predictors (e.g., SNPs). Other regularization methods related to RR are able to select a subset of predictors from a large set of predictors. Examples of such methods are the* least absolute shrinkage and selection operator* (LASSO), group LASSO [18], adaptive LASSO [19], and the elastic net [20].

In a GWAS, SNP selection is a desirable property when trying to find regions in the DNA that bear a causal influence on the outcome. However, there is mixed evidence for the claim that selection techniques in general improve the overall predictive accuracy of the polygenic score. Some studies suggest that preselection of markers (e.g., SNPs), based on either linkage disequilibrium or (in-sample) univariate association results, is detrimental to predictive accuracy (e.g., [3, 8, 11, 21]). Moreover, there is no conclusive evidence on the relative performance of RR-type methods and LASSO-type methods. For instance, using a simulation study, Ogutu et al. [22] find that LASSO-type methods outperform classic RR, whereas other studies find that RR outperforms LASSO and similar variable selection methods (e.g., [23–25]). A reasonable proposition is that the relative performance of RR and LASSO depends on trait architecture (e.g., [21, 26]). In particular, a low number of causal SNPs favor LASSO-type methods, whereas an intermediate or high number of causal variants favor RR-type methods. Regularization methods performing selection are computationally more involved and less amenable to incorporate nonlinear SNP effects than RR. For the above reasons, as well as our aim to provide a clear overview of RR, we focus in this paper primarily on RR.

The remainder of this paper is organized as follows. In Section 2, we present the theory underlying RR. In Section 3, we show that RR can be perceived as a method between OLS and RSR, leveraging the advantages of these two methods. Subsequently, in Section 4, we discuss the relation between RR and the best linear unbiased prediction used in animal breeding and the relation between RR and LASSO-type methods. In Section 5, we pay special attention to the effect standardization of SNP data has on the implicit assumptions about the genetic architecture of traits. As indicated, the feasibility of RR depends critically on the use of computationally efficient approaches. These will be discussed in Section 6. Related to this, in Section 7, we will discuss methods to tune the penalty parameter of RR. Following that, in Section 8, advanced RR techniques will be discussed, such as modelling nonlinear effects using RR, weighting SNPs differently, and incorporating information from earlier studies.

In order to assess the current and future use of ridge regression for prediction in quantitative genetics, we run a suite of simulations. The design of the simulations and the results are presented in Section 9. Based on these results we will estimate the effect sample size, the number of SNPs, the number of causal SNPs, and trait heritability have on the predictive accuracy of RR and the classical RSR approach. Using these estimates we will extrapolate how RR and RSR are expected to perform relative to each other in large scale studies (e.g., *N* ≥ 100,000). Finally, in Section 10, we summarize the most important aspects of RR in the context of prediction in quantitative genetics and discuss our expectations for its future uses.

## 2. Ridge Regression

Using ridge regression (RR) for prediction in quantitative genetics was first proposed by Whittaker et al. [27]. RR can be understood as follows. Like regular* least-squares* methods RR minimizes a loss function that includes the sum of squared regression residuals. However, opposed to least squares, the loss function also includes a term consisting of positive penalty parameter *λ* times the model complexity, measured by the sum of squared regression weights [9]. This penalty prevents overfitting by shrinking the weights towards zero, ensuring that, even in case of multicollinearity and *P* ≫ *N*, the estimator has a solution. The RR estimator has a simple analytical solution.

More formally, given a set of *N* individuals, *P* SNPs, and *K* confounders, a linear model for quantitative outcome vector **y** (*N* × 1), with a matrix of SNP data **X** (*N* × *P*), and a matrix of confounders **Z** (*N* × *K*) as predictors, is given by

(1) $$\begin{array}{c} y=X\beta +Z\gamma +\varepsilon , \end{array}$$

where **β** is the vector of SNP effects, **γ** the vector of effects of the confounders, and **ε** the phenotype noise.

In this particular case, we consider a large set of SNPs and a small set of potential confounders. Since one of our aims is to prevent any spurious relations via the confounders, we use a loss function that does not apply shrinkage to these. Therefore, the RR estimator minimizes

(2) $$\begin{array}{c} L_{\text{RR}}\left(\beta ,\gamma \right)={\left(y-X\beta -Z\gamma \right)}^{⊤}\left(y-X\beta -Z\gamma \right)+\lambda \beta^{⊤}\beta . \end{array}$$

Under this loss function, the RR estimator of **β** is given by

(3) $$\begin{array}{c} {\hat{\beta }}_{\text{RR}}={\left(X^{⊤}M_{Z}X+\lambda I\right)}^{-1}X^{⊤}M_{Z}y, \end{array}$$

where **M**_**Z**_ = **I** − **Z**(**Z** ^*⊤*^ **Z**)^−1^ **Z** ^*⊤*^ is the projection matrix, removing the effects of the confounding variables. The larger the *λ* is, the more the shrinkage is applied. When *λ* = 0, RR corresponds to OLS. The OLS estimator only exists if rank(**X** ^*⊤*^ **M**_**Z**_ **X**) = *P*, meaning that there is no perfect collinearity amongst the SNPs and that *P* ≤ *N*. However, in a GWAS, almost invariably *P* ≫ *N*. Therefore, OLS cannot be applied in this context. However, the RR estimator has a solution for any *λ* > 0, even if *P* ≫ *N*.

*Heteroskedastic ridge regression* (HRR) is a generalization of RR, where each SNP *p* receives a different amount of shrinkage, *λ*_*p*_ ≥ 0. The loss function of HRR is given by

(4) $$\begin{array}{c} L_{\text{HRR}}\left(\beta ,\gamma \right)={\left(y-X\beta -Z\gamma \right)}^{⊤}\left(y-X\beta -Z\gamma \right)+\lambda \beta^{⊤}\Lambda \beta , \end{array}$$

where Λ = diag(*λ*_1_,…, *λ*_*P*_). The corresponding estimator is given by

(5) $$\begin{array}{c} {\hat{\beta }}_{\text{HRR}}={\left(X^{⊤}M_{Z}X+\lambda \Lambda \right)}^{-1}X^{⊤}M_{Z}y. \end{array}$$

The *P* × *P* matrix **X** ^*⊤*^ **M**_**Z**_ **X** in (3) and (5) can be regarded as a map of the estimated correlation (linkage disequilibrium) between markers. OLS takes this linkage disequilibrium fully into account at the expense of overfitting the data, whereas RSR completely ignores it. For this reason, when constructing a polygenic score, RSR is often used in combination with a heuristic procedure, known as linkage disequilibrium pruning, which selects SNPs that are not too strongly correlated. As is shown in the next section, RR leverages the two extremes of OLS and RSR. Therefore, opposed to RSR, RR does not require the* a priori* selection of SNPs; RR is able to handle linkage disequilibrium between markers [10, 11].

RR is expected to perform particularly well under a scenario where a substantial proportion of the SNPs is expected to contribute to the phenotype and where each contribution is small.

## 3. The Limiting Cases of Ridge Regression

Varying the penalty weight, *λ*, allows specifying special cases of RR. Prediction by RR can be perceived as a method that lies between prediction based on OLS estimates considering all SNPs jointly and OLS estimates considering each SNP separately. By definition of RR [9], for sufficiently low shrinkage, the RR estimates converge to the multiple regression estimates [10], provided these are unique. For sufficiently high shrinkage a RR prediction score is equivalent to an RSR prediction score, in terms of the proportion of variance accounted for by the respective scores. For ease of notation, we assume in this section that there are no confounders **Z**.

To establish the aforementioned relations, two conditions are needed. First, the measure of predictive accuracy is independent of scale. That is, given an out-of-sample quantitative outcome vector (**y**_2_) and its prediction $\left({\hat{y}}_{2}\right)$, the accuracy measure should be such that for any coefficient *b* > 0 the accuracy of prediction ${\hat{y}}_{2}$ is identical to that of prediction ${\hat{y}}_{2}^{*}=b{\hat{y}}_{2}$. An example of such a measure is the *R* ^2^ of an outcome and its prediction. The second condition is that SNP data are standardized, such that each SNPs *p* has mean zero (**x**_*p*_^*⊤*^ **ι** = 0, where **ι** ^*⊤*^ = (1,…, 1)) and equal standard deviation (**x**_*p*_^*⊤*^ **x**_*p*_ = *c*, where *c* is a scalar).

Consider the prediction of **y**_2_ based on *N*_2_ × *P* out-of-sample genotype matrix **X**_2_, using in-sample RR estimates ${\hat{\beta }}_{\text{RR}}$. This prediction is given by ${\hat{y}}_{2}=X_{2}{\hat{\beta }}_{\text{RR}}$. Based on the first condition, we can multiply the prediction ${\hat{y}}_{2}$ by *b* = (1 + *λ*). This is equivalent to inflating the RR estimates by (1 + *λ*) instead of inflating the predictions. Thus, we can take ${\hat{\beta }}_{\text{RR}}^{*}=(1+\lambda ){\hat{\beta }}_{\text{RR}}$. This yields

(6) $$\begin{array}{c} {\hat{\beta }}_{\text{RR}}^{*}={\left(\alpha I+(1-\alpha )X^{⊤}X\right)}^{-1}X^{⊤}y, \end{array}$$

where *α* = (1 + *λ*)^−1^ *λ* ∈ (0,1). The OLS estimator considering all SNPs jointly is given by

(7) $$\begin{array}{c} {\hat{\beta }}_{\text{OLS}}={\left(X^{⊤}X\right)}^{-1}X^{⊤}y. \end{array}$$

Thus, it follows that when *α* goes to zero (i.e., *λ* goes to zero), the RR estimator goes to the OLS estimator. Moreover, as *α* goes to one (i.e., *λ* becomes sufficiently large), the inflated RR estimator goes to **X** ^*⊤*^ **y**.

Using the condition of having standardized SNPs, we can rewrite the RSR for SNP *p* as ${\hat{\beta }}_{p}=x_{p}^{⊤}y$, where **x**_*p*_ is the standardized genotype vector of SNP *p*. This expression can be vectorized over all SNPs as ${\hat{\beta }}_{\text{RSR}}=X^{⊤}y$. From this, it follows that the inflated RR estimates approach the RSR estimates as *λ* becomes sufficiently large.

## 4. Related Methods

Prediction using RR is related to the predictions that arise under a widely used simple mixed linear model, commonly referred to as the* animal model*. In such a model, expected genetic relatedness is mapped to phenotypic relatedness. Usually pedigree information is used to infer genetic relatedness. However, with the advent of genome-wide molecular data, mixed models that use SNPs to estimate genetic relatedness have been proposed (e.g., see Yang et al. [28]). In most mixed models using SNPs, the prior assumption is that SNP effects are normally distributed with mean zero and variance *σ*_**β**_^2^, and the error terms in the phenotype are also normally distributed with variance *σ*_**ε**_^2^.

To understand the relation between RR and mixed models, consider the following mixed linear model

(8) y=Xβ+Zγ+ε,β~N0,σβ2IP,ε~N0,σε2IN,

where *σ*_**β**_^2^ is the SNP effect variance and *σ*_**ε**_^2^ the noise variance. In this model the effects of the confounders, **Z**, are assumed to be fixed. For the remainder of this section we ignore the confounders for ease of notation. The parameters *σ*_**ε**_^2^ and *σ*_**β**_^2^ can be estimated using, for instance,* maximum likelihood*,* restricted maximum likelihood* [29], or* expectation maximization* [30]. Alternatively, these parameters can be fixed by using prior information from other data sets; see, for instance, Hofheinz et al. [31].

Consider conditional expectations *𝔼*[**β**∣**y**] and *𝔼*[**y**_2_∣**y**]. In a mixed linear model such expectations are known as the best linear unbiased prediction (BLUP) [32–36]. BLUP was first proposed by Henderson [32] in order to obtain estimates of the so-called* breeding values*, that is, the part of the phenotype that can be attributed to genetic variation.

Provided that the RR penalty *λ* = *σ*_**ε**_^2^/*σ*_**β**_^2^, the BLUP of SNP effects [28, 37, 38] is equivalent to the RR estimator. Under that same condition, the BLUP of the SNP-based breeding values is equivalent to RR prediction. Such* genomic estimated breeding values* [38] contain the part of the phenotype that can be attributed to the genetic variation in the genotyped markers.

To understand this equivalence, first we rewrite the RR estimator in (3). By applying the* Sherman-Morrison-Woodbury formula* [39, 40] to the *P* × *P* inverse of **X** ^*⊤*^ **X** + *λ* **I**, we obtain

(9) β^RR=1λIP−X⊤XX⊤+λIN−1XX⊤y=1λX⊤IN−XX⊤+λIN−1XX⊤y=1λX⊤XX⊤+λIN−1XX⊤+λIN−XX⊤y=X⊤XX⊤+λIN−1y.

Second, by rewriting (8) in terms of the joint distribution of **y** and **β**:

(10) $$\begin{array}{c} \left(\begin{array}{c} y\\ \beta \end{array}\right)\sim N\left(\left[\begin{array}{c} 0\\ 0 \end{array}\right],\left[\begin{array}{cc} \sigma_{\beta }^{2}XX^{⊤}+\sigma_{\varepsilon }^{2}I_{N} & \sigma_{\beta }^{2}X\\ \sigma_{\beta }^{2}X^{⊤} & \sigma_{\beta }^{2}I_{P} \end{array}\right]\right), \end{array}$$

the BLUP of **β** is given by the expectation of **β** conditional on **y** [17]. This yields

(11) β^BLUP=σβ2X⊤σβ2XX⊤+σε2IN−1y=X⊤XX⊤+σε2σβ2IN−1y.

Clearly, when *λ* = *σ*_**ε**_^2^/*σ*_**β**_^2^, ${\hat{\beta }}_{\text{RR}}={\hat{\beta }}_{\text{BLUP}}$.

In addition, from a Bayesian perspective the posterior mode of the distribution of SNP effects (i.e., the mode of the distribution conditional on a training set) can also be used as point estimator. Estimation using the posterior mode is known as maximum a posteriori (MAP) estimation. However, due to the normality of **β** and **ε** the mode coincides with the conditional expectation *𝔼*[**β**∣**y**]. Therefore, MAP estimation of **β** in (8) is equivalent to BLUP.

**Figure 1:**
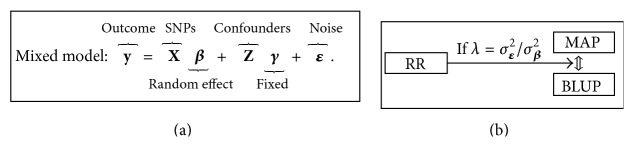
Diagram (b) showing the relation between estimation of SNP effects using* ridge regression* (RR), the* best linear unbiased prediction* (BLUP), and* maximum a posteriori* (MAP) estimation, under the specified* mixed linear model* (a).

Consequently, there exists a *λ* such that the RR estimator of SNP effects is equivalent to its BLUP [16, 41] and by extension to the MAP estimator. The diagram in Figure 1 summarizes the relations between RR, BLUP, and MAP.

### 4.1. SNP Selection Using LASSO-Type Methods

An important feature that RR lacks is the selection of SNPs. LASSO-type methods, such as the LASSO, group LASSO, adaptive LASSO, and the elastic net, are able to select SNPs. The key to achieving SNP selection is to include an *L*_1_ penalty, that is, adding a penalty consisting of a penalty parameter, *λ*, times ‖**β**‖_1_ = |*β*_1_ | +⋯+|*β*_*P*_|. The loss function of the LASSO is given by

(12) $$\begin{array}{c} L_{\text{LASSO}}\left(\beta ,\gamma \right)={\left(y-X\beta -Z\gamma \right)}^{⊤}\left(y-X\beta -Z\gamma \right)+\lambda {\left\|\beta \right\|}_{1}. \end{array}$$

This function is highly similar to the RR loss function in (2). The most important property of the LASSO is that it performs variable selection; that is, for a sufficiently large *λ* many of the SNP coefficients *β*_*p*_ will be zero. The higher the *λ* is, the fewer the nonzero SNP effects are obtained by the LASSO. Moreover, this method also shrinks the nonzero coefficients, that is, the estimated effects of the selected SNPs.

The loss function of the elastic net [20] is obtained by taking a convex combination of **β** ^*⊤*^ **β** and ‖**β**‖_1_ as penalty; that is,

(13) Lnetβ,γ=y−Xβ−Zγ⊤y−Xβ−Zγ+λαβ⊤β+(1−α)β1,

with *λ* ≥ 0 and *α* ∈ [0,1]. The elastic-net method preserves SNP selection, while allowing more than *N* of *P* SNPs to be selected. Taking a convex combination of the two norms hardly increases the computational costs of solving this problem, when compared to solving the LASSO problem [20]. Typically, the LASSO solution is obtained by means of the least-angle regression algorithm [42]. This algorithm entails an iterative procedure, where at most one SNP can enter the model at a time. Therefore, LASSO-type methods are computationally far more involved than RR-type methods.

Finally, the group LASSO [18] splits the *P* predictors in *G* mutually disjoint groups, with *p*_*g*_ predictors in group *g*, and associated effects **β**_*g*_, for groups *g* = 1,…, *G*. The group LASSO minimizes

(14) Lgroupβ,γ=y−Xβ−Zγ⊤y−Xβ−Zγ+λ∑g=1Gβg⊤βg.

Each group can be chosen, for instance, to represent a single gene in terms of its SNPs. The group LASSO induces sparsity at the group level (e.g., a gene is either included as a whole or wholly excluded), whereas within a group the individual regressors receive an *L*_2_ penalty. To the best of our knowledge, Sabourin et al. [43] provide the first, and so far only, application of a (modified) group LASSO using SNP data to construct polygenic scores. In this study, each SNP is considered as a group, with two effects: an additive and a dominance effect. In a simulation with mild to strong dominance, this method improves accuracy, compared to an RSR-type approach [43]. For a detailed comparison of LASSO-type methods and RR, we refer to Hastie et al. [44].

## 5. The Implications of Standardizing SNPs

In the preceding sections, we have only considered SNP standardization as a tool to show that RR can be perceived as a method between the classical GWAS approach and the OLS approach considering all SNPs jointly. However, SNP standardization is often used in the mixed linear model in (8).

The reason for this is that standardization has a profound effect on the implicit assumptions about the effect sizes of SNPs. We show in this section that the standardization we use is equivalent to HRR applied to raw genetic data, where SNPs measuring rare variants receive less shrinkage than SNPs measuring common variants.

More specifically, let **G** (resp., **G**_2_) denote raw SNP data in sample (out of sample) that has already been mean-centered but not yet standardized to have the same variance. The standardized data **X** in Section 3 can now be obtained by postmultiplying **G** by a diagonal matrix **D**. That is, **X** = **G** **D**, where

(15) $$\begin{array}{c} D=\mathrm{diag}\left({\left\{\sqrt{\frac{N-1}{x_{p}^{⊤}x_{p}}}\right\}}_{p=1,\ldots ,P}\right). \end{array}$$

Under the reasonable assumption that only SNPs are considered for which in-sample variation occurs, this matrix **D** is invertible.

By applying this transformation in both the training and test set, RR prediction based on standardized data is given by

(16) y^2=X2X⊤X+λI−1X⊤y=G2DDG⊤GD+λI−1DG⊤y=G2G⊤G+λD−2−1G⊤y.

This shows that RR applied to standardized SNP data is equivalent to HRR, with Λ = **D** ^−2^, applied to raw genotype data. Here, the SNP-specific shrinkage depends on the amount of SNP variation. This type of shrinkage implicitly assumes that the standardized SNPs have homoskedastic effects, whereas the underlying raw genotypes (i.e., the count data) have effects of which the variance decreases with minor allele frequency. That is, rare alleles are assumed to have larger effects on average than common variants. For a qualitative treatment of the relation between allele frequency and expected effect sizes, see, for instance, Manolio et al. [45].

To be more precise, this type of shrinkage corresponds to the implicit assumption that the variance of the effect of raw SNP *p*, denoted by *σ*_**β**_*p*__^2^, with allele frequency *f*_*p*_, is proportional to (2*f*_*p*_(1 − *f*_*p*_))^−1^. This assumption implies that when *f*_*p*_ is close to one or zero, the variance of the effect size is expected to be large, whereas for *f*_*p*_ close to 50% the variance of the effect size attains its minimum.

Naturally, raw SNP effect variances responding differently to allele frequency can be conceived. As indicated by Manolio et al. [45] such relations depend on the effect of the trait under consideration, on the fitness of the individual. Therefore, a natural extension would be to consider HRR with Λ = **D** ^*α*^. Here *α* = 0 corresponds to a trait for which allele frequency is independent of effect size and *α* = −2 corresponds to the relation described before. Moreover, −2 < *α* < 0 describes a trait for which there is a slight relation between allele frequency and effect size. It is interesting to note that *α* > 0 corresponds to a trait where diversity is an asset, that is, a trait in which variants causing phenotypic divergence between individuals tend to become common. Finally, *α* < −2 would correspond to a trait for which there has been strong selection pressure causing convergence; only very rare variants are expected to have a large effect. Thus, in future work *α* can be considered as an additional hyperparameter which might boost predictive accuracy and of which the estimate would reveal something about the selection pressure regarding the trait under consideration. The same type of transformation has been proposed by Speed et al. [46] for improving estimation of SNP-based heritability in a mixed linear model.

## 6. Computational Costs

The main hurdle in computing RR predictions is estimating the *P* parameters, when *P* ≫ *N*. In particular, a naive approach requires solving a system with *P* unknowns. However, RR can be implemented in a computationally efficient way. When *P* > *N*, using dimensionality reduction techniques the complexity of RR can be reduced from *𝒪*(*P* ^3^) to *𝒪*(*PN* ^2^) in case one is interested in the estimated effects [47].

Moreover, if the focus lies solely on obtaining predictions, a nonparametric representation of RR reveals the fact that a dual formulation exists, which can be perceived as solving a linear model with *N* unknowns [48]. Solving such a system has a complexity slightly less than *𝒪*(*N* ^3^). Building on this computationally efficient approach, RR can also efficiently control for confounders, both in sample and out of sample.

Finally, when considering a wide array of values of *λ*, RR can be reformulated to generate predictions for all values of *λ* jointly by exploiting the properties of the eigendecomposition of an *N* × *N* matrix, thereby yielding a complexity of *𝒪*(*N* ^3^).

To understand these reductions in computational costs, consider the RR estimator in (9), used to show equivalence of RR and the BLUP. Premultiplying this expression by **X**_2_, the out-of-sample prediction is given by

(17) $$\begin{array}{c} {\hat{y}}_{2}=X_{2}X^{⊤}{\left(XX^{⊤}+\lambda I_{N}\right)}^{-1}y. \end{array}$$

As discussed, accounting for confounding variables is important. Let **Z** be the in-sample *N* × *K* matrix of confounders and **Z**_2_ the out-of-sample *N*_2_ × *K* matrix of confounders. By replacing **X** by **X** ^∗^ = **M**_**Z**_ **X** and **X**_2_ by **X**_2_^∗^ = **M**_**Z**_2__ **X**_2_, where **M**_**C**_ is the projection matrix removing the effects of **C**, we find that

(18) $$\begin{array}{c} {\hat{y}}_{2}=A_{21}^{*}{\left(A^{*}+\lambda_{\text{GRM}}I_{N}\right)}^{-1}y, \end{array}$$

where **A** ^∗^ = **M**_**Z**_ **A** **M**_**Z**_ and **A**_21_^∗^ = **M**_**Z**_2__ **A**_21_ **M**_**Z**_, **A** = *P* ^−1^ **X** **X** ^*⊤*^ and **A**_21_ = *P* ^−1^ **X**_2_ **X** ^*⊤*^, and *λ*_GRM_ = *P* ^−1^ *λ*. Therefore, one can correct for covariates by simply pre- and postmultiplying *N*_(2)_ × *N* matrices, by appropriate projection matrices.

Matrices **A** and **A**_21_ both have the interpretation of a SNP-based* genetic relationship matrix* (GRM) [28], measuring the genetic similarity of individuals in the space of additive SNP effects.

Given the eigendecomposition

(19) $$\begin{array}{c} A^{*}=Q\mathrm{diag}\left({\left\{\theta_{i}\right\}}_{i=1,\ldots ,N}\right)Q^{⊤}, \end{array}$$

RR prediction can be written as

(20) $$\begin{array}{c} {\hat{y}}_{2}=A_{21}^{*}Q\mathrm{diag}\left({\left\{\frac{1}{\theta_{i}+\lambda_{\text{GRM}}}\right\}}_{i=1,\ldots ,N}\right)Q^{⊤}y. \end{array}$$

If *P* ≫ *N*, this approach is far more efficient than the naive approach to RR prediction. GRMs can be computed efficiently in packages such as  PLINK 1.9 [49] and  GCTA [28]. The most involved step in the prediction procedure is finding the eigendecomposition of **A** ^∗^.

## 7. Tuning and Interpreting *λ*

So far, it was assumed that the penalty strength parameter *λ* is given. However, in most applications of RR the optimal *λ* is not known in advance. Here, we discuss three ways for choosing *λ*.

The dominant approach in the machine learning literature for tuning *λ* is by maximizing out-of-sample predictive accuracy of RR using* cross-validation* (CV). In CV one considers a fine grid *ℒ* of potential values of *λ*. The data are randomly split in a (small) test set (e.g., 10% of the sample) and CV set (90%). To the CV set one applies *K*-fold CV (e.g., *K* = 10), meaning that one splits the CV sample randomly in *K* blocks of (approximately) equal size. In each fold *K* − 1 blocks are considered as CV training set and the remaining block as CV test set. Using RR for all values of *λ* ∈ *ℒ*, predictions in the CV test set are generated. Each block is the CV test set precisely once. After the *K*-folds, the predictive accuracy over all CV test sets is evaluated for all *λ* ∈ *ℒ*. Now, $\hat{\lambda }$ is set to maximize the cross-validation accuracy. Finally, using $\hat{\lambda }$ the predictive accuracy in the final test is considered, using the full CV set as training data. For a more detailed treatment of CV, see, for instance, Hastie et al. [44].

*Nested cross-validation* (NCV) is a natural extension of CV, where the sample is randomly split in *S* “super”-blocks of approximately equal size (e.g., *S* = 10) and where there are *S* “super”-folds. In each superfold, one block is considered as final test set and *S* − 1 other blocks as CV set. To this CV set and test set one applies regular *K*-fold CV. Each superblock is used as final test set precisely once.

Classical CV is used to fit the model and to assess its predictive accuracy; one can judge the merits of a set of values of the hyperparameter by means of the CV procedure and apply the optimal value to a new part of the sample which has not yet been considered. Using NCV one can test whether the hyperparameter and accuracy that result from classical CV are robust; NCV can show the amount of variation in either of these over the “super”-folds.

CV requires a computationally efficient strategy since a different set of RR predictions will result for each different value of *λ*. However, a large set of different values of *λ* can be evaluated in one step at nearly the same costs of evaluating a single value of *λ*. This approach avoids computing a full RR solution for each *λ* separately. To see this, the formulation of RR prediction in (20) is highly relevant. In this equation, the eigendecomposition of **A** ^∗^ is independent of *λ*. Thus, predictions for each *λ* ∈ {*λ*_1_,…, *λ*_*L*_} can be obtained by the following equation:

(21) Y^2=A2,1∗Q·θ1+λ1−1⋯θ1+λL−1⋮⋱⋮θN+λ1−1⋯θN+λL−1∘Q⊤yι⊤,

where **ι** ^*⊤*^ = (1,…, 1) and “∘” denotes the element-wise (Hadamard) product. A  MATLAB implementation of this approach to RR prediction is provided in Algorithm 1. The computation of the eigendecomposition of **A** ^∗^ has a computational complexity of *𝒪*(*N* ^3^). Given this decomposition, the prediction consists of (*N*_2_ + 3)*NL* + (*L* + 1)*N* ^2^ simple operations such as multiplication and addition of scalars.

**Figure 2:**
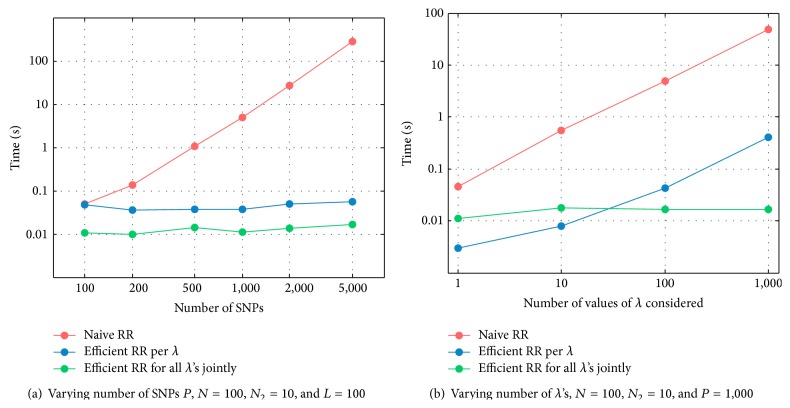
CPU time in seconds of prediction using naive RR (red), efficient RR for each *λ* separately (blue), and efficient RR considering all *λ*'s jointly (green).

To illustrate the differences in the respective approaches to RR, Figure 2 shows the CPU time for (i) the naive approach in (3) which involves solving *P* unknowns, (ii) the dual formulation in (18) which requires solving *L* systems with *N* unknowns each, and (iii) the dual formulation in (21) solving for all values of *λ* jointly. These results are obtained by applying the approaches to simulated data, with baseline settings *N* = 100, *N*_2_ = 10, *P* = 1000, and *L* = 100, and by varying the levels of the factors *N* and *L*, one factor at a time. In order to ensure no approach has an advantage in terms of preprocessing of the data (e.g., constructing *P* ^−1^ **X** **X** ^*⊤*^ and its eigendecomposition) all reported CPU times include these preprocessing steps.

In Figure 2(a), we see that as the number of SNPs *P* increases the time required by the naive approach keeps growing at a fixed rate, whereas the time required by the dual approaches remains unchanged. Moreover, the approach considering all values of *λ* jointly outperforms the dual approach solving *L* separate systems. When sample size *N* is relatively large compared to *P* the dual formulations lose their advantage compared to the naive approach. This is not surprising: when *N* > *P* the dual formulation requires solving more unknowns than the naive approach. Concordantly, when faced with data in which *N* ≤ *P* one can apply the dual approach, and when *N* > *P* one can use the classical approach to RR.  Figure 2(b) shows that for a very small set of *λ*'s the dual formulation solving *L* systems with *N* unknowns is faster than the formulation solving for all values of *λ* jointly. However, the CPU time required by the former approach increases continuously with *L*, whereas the CPU time of the method considering all *λ*'s jointly hardly changes. When *L* ≥ 10, the latter method attains a better CPU time than the former method does.

The second method for setting *λ* is based on the mixed model in (8). In this model, the optimal hyperparameter is a function of *σ*_**ε**_^2^ and *σ*_**β**_^2^. Therefore, one can estimate the mixed linear model using methods such as (restricted) maximum likelihood [28, 29] and take *λ* = *σ*_**ε**_^2^/*σ*_**β**_^2^.

Finally, one can use an existing heritability estimate of the trait under consideration. Given the following definition of SNP-based heritability,

(22) $$\begin{array}{c} h_{\text{SNP}}^{2}=\frac{P\sigma_{\beta }^{2}}{P\sigma_{\beta }^{2}+\sigma_{\varepsilon }^{2}}, \end{array}$$

provided the SNP data are standardized as *Z*-scores, it is shown by Hofheinz et al. [31] that the RR shrinkage parameter *λ* can be written as a function of the SNP-based heritability. Specifically, simple algebra shows that, under the above definition of SNP-based heritability,

(23) $$\begin{array}{c} \lambda =P\left(\frac{1}{h_{\text{SNP}}^{2}}-1\right). \end{array}$$

This implies that heritability estimates can be used to set *λ* [31]. When using a GRM (*P* ^−1^ **X** **X** ^*⊤*^) to carry out RR prediction, the corresponding shrinkage parameter *λ*_GRM_ = *P* ^−1^ *λ*. This implies the relation between *λ*_GRM_ and *h*_SNP_^2^ is given by *λ*_GRM_ = (*h*_SNP_^2^)^−1^ − 1.

## 8. Advanced Ridge Regression Methods

### 8.1. Heteroskedastic Ridge Regression

A point of critique regarding the use of RR is the lack of SNP selection. However, for highly polygenic traits, given current sample sizes, there is evidence that SNP selection is sometimes detrimental to predictive accuracy (e.g., [3, 8, 21]). Nevertheless, since RR can be used for inference just as well as RSR, the approach of selecting SNPs that attain a *p* value below some threshold *τ* in the GWAS can also be extended to RR.

In a spirit similar to that of SNP selection, one can argue in favor of a heteroskedastic ridge regression (HRR), where each SNP receives a different amount of shrinkage [50, 51]. As with homoskedastic shrinkage, this SNP-specific shrinkage might be based on either results from the training set or prior information from different data sets. Depending on the size of SNP-specific shrinkage, this method can leverage between SNP selection and full inclusion. Based on prior evidence or in-sample evidence the weight assigned to a SNP can be made arbitrarily small or arbitrarily large given the amount of evidence for association with the outcome. SNP-specific shrinkage opens up the door for a whole array of HRR methods (e.g., [50, 51]).

The HRR estimator in (5) and resulting predictions can be rewritten as

(24) $$\begin{array}{c} {\hat{\beta }}_{\text{HRR}}=\Lambda^{-1}X^{⊤}{\left(X\Lambda^{-1}X^{⊤}+\lambda I\right)}^{-1}y, \end{array}$$

(25) $$\begin{array}{c} {\hat{y}}_{2}=X_{2}\Lambda^{-1}X^{⊤}{\left(X\Lambda^{-1}X^{⊤}+\lambda I\right)}^{-1}y, \end{array}$$

where Λ = diag({*λ*_*p*_}_*p*=1,…,*P*_) is a diagonal matrix with SNP-specific shrinkage effects.

It is implied by (24) and (25) that HRR can be carried out using the same machinery as homoskedastic RR, by first weighting the SNPs appropriately. More specifically, take **X** ^∗^ = **X**Λ^1/2^ and **X**_2_^∗^ = **X**_2_Λ^1/2^ and construct corresponding weighted GRMs by taking

(26) $$\begin{array}{c} A^{*}=M_{Z}\left(\frac{1}{P}X^{*}X^{*⊤}\right)M_{Z},\\ A_{21}^{*}=M_{Z_{2}}\left(\frac{1}{P}X_{2}^{*}X^{*⊤}\right)M_{Z}. \end{array}$$

Now, using the eigendecomposition defined in (19) of the weighted GRM defined in (26) and by subsequently applying (21) to resulting eigenvectors in **Q** and eigenvalues, {*θ*_*i*_}_*i*=1,…,*N*_, we obtain efficient out-of-sample HRR predictions.

### 8.2. Incorporating Information from Earlier Studies

Using HRR prediction it is possible to include results from a GWAS in other samples as prior information. Consider SNP-specific shrinkage, given by *λ*_*p*_ = *σ*_**ε**_^2^/*σ*_*β*_*p*__^2^, and a set of GWAS *t*-test statistics from another study without the presence of confounding variables. Given that ${\hat{\sigma }}_{\varepsilon }$ is approximately constant over the SNPs in the GWAS, the *t*-test statistic of SNP *p* can be written as

(27) $$\begin{array}{c} t_{p}\approx \frac{1}{{\hat{\sigma }}_{\varepsilon }}\left(\frac{x_{p}^{⊤}}{\sqrt{x_{p}^{⊤}x_{p}}}\right)y=\frac{1}{{\hat{\sigma }}_{\varepsilon }}x_{p}^{*⊤}y=\frac{1}{{\hat{\sigma }}_{\varepsilon }}{\hat{\beta }}_{p}, \end{array}$$

where **x**_*p*_^∗^ denotes SNP *p* standardized to unit length and ${\hat{\beta }}_{p}$ the estimated effect of the standardized SNP. It follows from this equation that these statistics are proportional to the estimated effects of standardized SNPs. Therefore, the square *t*-test statistics are approximately proportional to the square standardized GWAS estimates. Now, under the prior probability distribution that *β*_*p*_ ~ *𝒩*(0, *σ*_*β*_*p*__^2^) we have that ${\hat{\beta }}_{p}^{2}$ is a consistent estimator of *σ*_*β*_*p*__^2^. Correspondingly, the square *t*-test statistics are proportional to this estimator of the SNP-specific effect variance. Therefore, for a suitable choice of *λ* a consistent estimator of *λ*_*p*_ is given by $\lambda t_{p}^{-2}=\sigma_{\varepsilon }^{2}/{\hat{\beta }}_{p}^{2}$. In the framework of HRR, this entails setting $\hat{\Lambda }=\mathrm{diag}({\left\{t_{p}^{-2}\right\}}_{p=1,\ldots ,P})$. This definition of $\hat{\Lambda }$ implies that SNPs are weighted according to *t*_*p*_. From these weighted SNPs we can construct the weighted GRM and apply (25) to obtain out-of-sample HRR predictions which incorporate information from a GWAS in another dataset.

### 8.3. Nonlinear Prediction Methods

An important question in genetics is how nonlinear effects (e.g.,* dominance* and* epistasis*) contribute to the variation of complex traits. RR can efficiently implement such nonlinear SNP effects using the* kernel trick* from machine learning. Resulting* kernel ridge regression* (KRR) extends the nonparametric approach to RR, where genetic “similarities” in the space of additive effects are replaced by genetic “similarities” in a larger (potentially infinite) feature space, for instance, including two- or three-way interactions.

The efficient RR predictions in (17) are in essence a weighted average of the observed phenotypes in the training set. Weights are based on the genetic similarity of individuals in the test set and the training set. The more genetically similar two individuals are in the test and training set, the more weight will be given to the phenotype of the similar individual in the training set.

Classical RR measures genetic similarity of individuals in the space of additive effects and assigns weights accordingly. KRR, however, can measure genetic similarity in the space of more than just additive effects. This extended space can include, for instance, *d*-way interactions between SNPs. Now, a GWAS estimating all potential *d*-way interactions between SNPs is not feasible. However, with KRR, rather than having to estimate all coefficients of all nonlinear combinations of regressors, one can instead obtain the measure of genetic similarity in this higher-dimensional space by applying a simple kernel function *k*(**x**_*i*_, **x**_*j*_) to any two genotype vectors **x**_*i*_ and **x**_*j*_ corresponding to individuals *i* and *j*.

In this context, classical RR corresponds to *k*(**x**_*i*_, **x**_*j*_) = **x**_*i*_^*⊤*^ **x**_*j*_. Similarly, a function measuring similarity in the space consisting only of two-way linear interactions is given by

(28) $$\begin{array}{c} k\left(x_{i},x_{j}\right)={\left(x_{i}^{⊤}x_{j}\right)}^{2}. \end{array}$$

To see why this is so, consider expanding (28). We then have

(29) kxi,xj=∑p=1Pxipxjp2=∑p=1P ∑q=1Pxipxjpxiqxjq    =∑p=1P ∑q=1P(xipxiq)(xjpxjq)=ϕ(xi)⊤ϕ(xj),

where **ϕ**(**x**_*i*_)^*⊤*^ = ({{*x*_*ip*_ *x*_*iq*_}_*q*=1,…,*P*_}_*p*=1,…,*P*_). Thus, **ϕ**(**x**_*i*_) is a vector that contains all possible two-way interactions between the *P* markers. Kernel function *k*(**x**_*i*_, **x**_*j*_) represents the genetic similarity of individuals *i* and *j* in this space of all two-way interactions between SNPs.

The essence of KRR is the so-called kernel trick that allows one to efficiently compute the higher-dimensional similarity measure by applying a simple kernel function *k*(**x**_*i*_, **x**_*j*_) to any two input vectors for individuals *i* and *j* [52]. Provided the kernel is positive definite it constitutes the reproducing kernel of a unique* reproducing kernel Hilbert space* (RKHS) [53]. KRR then is equivalent to a so-called RKHS regression.

In the case of *d*-way interactions the associated kernel function *k*(**x**_*i*_, **x**_*j*_) can be evaluated for all pairs of individuals by raising each element of the GRM, *P* ^−1^ **X** **X** ^*⊤*^, to the power *d*. An alternative is the nonhomogeneous polynomial kernel of degree *d*, given by *k*(**x**_*i*_, **x**_*j*_) = (*c* + **x**_*i*_^*⊤*^ **x**_*j*_)^*d*^. This kernel, similar to the regular polynomial kernel of degree *d*, includes *d*-way interactions but also lower-order interaction terms including the “one-way interactions,” that is, simple additive linear effects.

The preceding example of the polynomial kernel of degree two shows how KRR can include dominance and epistasis in the prediction model. For frequently used kernels, such as the Gaussian (radial basis function) kernel, there exists a representation in which classical RR is applied to a model with infinitely many predictors, nevertheless yielding finite predictions. Obtaining the weights for infinitely many predictors is not possible. Hence, rather than aiming to obtain point estimates of **β**, KRR only aims to obtain predictions.

BLUP and, by extension, RR are special cases of prediction using KRR (e.g., [54, 55]). There has been a substantial amount of work in plant and animal breeding, aiming to improve predictive accuracy using KRR (e.g., [12, 15, 16]). A generally used kernel is the aforementioned Gaussian kernel, defined as

(30) $$\begin{array}{c} k\left(x_{i},x_{j}\right)=\exp\left[-\frac{d^{2}\left(x_{i},x_{j}\right)}{\eta }\right], \end{array}$$

where *d* ^2^(**x**_*i*_, **x**_*j*_) = (**x**_*i*_ − **x**_*j*_)^*⊤*^(**x**_*i*_ − **x**_*j*_) and hyperparameter *η* > 0. This type of kernel includes all conceivable linear interactions between the *P* SNPs and with themselves. Endelman [16] finds that the Gaussian kernel outperforms accuracy of RR and a Bayesian approach to LASSO, used to predict wheat and maize traits in samples, typically with about 300 observations and 3000 SNPs. Similarly, using a Bayesian approach, Crossa et al. [15] find in samples of about 250 observations, with 1100 SNPs, that both the Gaussian kernel and the LASSO outperform predictive accuracy of RR for grain yield and maize flowering traits. However, when comparing the LASSO with the Gaussian KRR, which of two the methods is better, depends on the trait. An efficient implementation of KRR based on maximum likelihood, using the Gaussian kernel, is available in the R package  rrBLUP [16].

Morota and Gianola [17] compare a wide range of kernels, such as the exponential [12, 16, 56], Matérn, diffusion (e.g., [57]), and *t* kernel [58], for the purpose of obtaining genomic estimated breeding values [38]. Though it is argued that selecting a suitable kernel is the most precarious step (e.g., [14]), current evidence suggests that most considered kernels attain a predictive accuracy similar to that of the Gaussian kernels [17]. Thus, it appears that the Gaussian KRR is a robust prediction method for quantitative traits, able to handle nonlinear genetic architectures. Moreover, Endelman [16] finds little evidence supporting the hypothesis that a Gaussian kernel is likely to overfit the data [56].

Given the current evidence, KRR using an appropriate kernel (e.g., the Gaussian kernel) is a promising prediction technique, especially for traits where epistatic effects and dominance are expected to contribute to trait variation. De los Campos et al. [14] suggest an interesting venue for further research on the use of KRR for prediction in quantitative genetics, by combining multiple kernels in a single model, each kernel representing a single variance component (e.g., additive, dominance, or epistasis). For a more detailed treatment of KRR and its uses in quantitative genetics, see Morota and Gianola [17].

Regarding the computation of predictions using KRR, let **K** denote the matrix of similarities in the higher-dimensional feature space in the training set, such that an element of this matrix *k*_*ij*_ is given by *k*(**x**_*i*_, **x**_*j*_) and let **K**_21_ be defined similarly for individuals in the test set versus individuals in the training set. Now, KRR prediction without confounders is given by ${\hat{y}}_{2}=K_{21}{\left(K+\lambda I\right)}^{-1}y$ and with confounders by

(31) $$\begin{array}{c} {\hat{y}}_{2}=M_{Z_{2}}K_{21}M_{Z}{\left(M_{Z}KM_{Z}+\lambda I\right)}^{-1}y, \end{array}$$

where, as before, **M**_**C**_ is the projection matrix removing the effects of **C**.

In the case of the nonhomogeneous polynomial kernel of degree *d*, given the GRMs, *P* ^−1^ **X** **X** ^*⊤*^ and *P* ^−1^ **X**_2_ **X** ^*⊤*^, the matrices **K** and **K**_21_ can be obtained efficiently by adding a constant *c* to each element of the GRMs and by raising each resulting element to the power *d*. When *c* > 0 and *d* ∈ {1,2,…} are not fixed, these are additional hyperparameters which can be tuned via (*N*)CV.

## 9. Simulation Study

An important question is under what circumstances can we expect RR to yield more accurate predictions than RSR? The answer to this question can help us assess the merits of RR in quantitative genetics. As discussed, prediction using RR is intimately related to the BLUP of the phenotype under a mixed linear model in which SNP effects are assumed to be all drawn from a normal distribution. This corresponds to idea of each SNP making a tiny contribution to phenotype. Therefore, it is reasonable to assume that RR will perform well when the SNP effects are as such. However, given that not all SNPs in existence are causally affecting the outcome, an open question is how does RR perform when only a subset of SNPs affects the outcome?

Moreover, an important factor influencing predictive accuracy of a classical polygenic score is the training sample size. Therefore, RR is likely also to be very sensitive to the sample size. Finally, the more heritable a trait is, the easier it should be to detect the effects of SNPs. Thus, an additional question is how do RR and RSR perform under different levels of heritability?

In short, we want to know the relative predictive accuracy of RR and RSR (i) for a wide range of trait architectures and (ii) under particular combinations of sample size and the number of genotyped SNPs. To answer this question we run a suite of simulations. In these analyses, we vary sample size of the training set (*N*), the number of genotyped SNPs (*P*), the fraction of SNPs exerting a causal influence (*f*_*C*_), and the SNP-based heritability (*h*_SNP_^2^).

Table 1 shows the levels we consider for these factors. In addition, a range of values for *λ* on the interval [10^−6^; 10^9^] is considered. Each unique combination of levels of these factors constitutes a scenario. The total number of scenarios is *S* = 7 × 12 × 37 × 20 = 62,160. We consider *R* = 21 runs, yielding *S* × *R* = 1,305,360 combinations of levels and runs.

For a combination of sample size, the number of SNPs, trait heritability, and a fraction of causal SNPs chosen from the levels shown in Table 1, let *C* be the corresponding number of causal SNPs. Now, the data generating process for this combination of levels is given by

(32) yi =∑p=1Cxipβp+εi, for  i=1,…,Ntotal,xip =gip−2fp2fp(1−fp), for  i=1,…,Ntotal, p=1,…,P,gip ~Binom2,fp, for  i=1,…,Ntotal, p=1,…,P,fp ~U0.05,0.95, for  p=1,…,P,βp ~N0,σβ2, for  p=1,…,P,εi ~N0,σε2, for  i=1,…,Ntotal,

where Binom(*a*, *b*) denotes the binomial distribution with *a* draws each with probability of success *b* and *𝒰*(*a*, *b*) denotes the uniform distribution on the interval (*a*, *b*). This data generating process corresponds to a quantitative trait which is normally distributed and has only additive genetic variation to which common variants contribute (i.e., minor allele frequency above 5%).

The total number of observations *N*_total_ includes the individuals in the test set. The size of the test set is 10% of the size of the training set, hence, yielding *N*_total_ = ⌊1.1*N*⌋. Here, ⌊*x*⌋ denotes the nearest smaller integer.

In order not to be dependent on a single generated dataset, the entire simulation consists of *R* = 21 independent runs (replications). In each run we simulate only one set of genotype data for *N*_max⁡_ = 22,000 individuals and *P*_max⁡_ = 500,000 SNPs. Given any combination of *N* and *P* listed in Table 1 we can take an appropriate submatrix of the genotype matrix. To this submatrix we apply a set of *P* weights of which some are zero, such that we attain the desired fraction of SNPs being causal. Moreover, by scaling these weights and the noise vector **ε** appropriately we can attain any specified heritability. The result is a four-dimensional phenotype array with individuals along the first dimension and the factors *P*, *f*_*C*_, and *h* ^2^ along the remaining dimensions.

When computing the out-of-sample predictions based on RR and RSR, the available genotype matrix only depends on *N* and *P*, not on *h* ^2^ or on *f*_*C*_. Therefore, given *N* and *P*, when *N* ≤ *P* the eigendecomposition of the *N* × *N* GRM, *P* ^−1^ **X** **X** ^*⊤*^, can be reused for all combinations of *h* ^2^ and *f*_*C*_. Moreover, the approach has already been amended to reuse the eigendecomposition for different values of *λ*. Similarly, when *N* > *P* the eigendecomposition of *P* × *P* matrix *P* ^−1^ **X** ^*⊤*^ **X** can be reused. Since there only are 7 unique levels of *N* and 12 unique levels of *P*, RR prediction (i) for the 62,160 scenarios per replication reduces to computing 7 × 12 = 84 eigendecompositions and (ii) for each scenario to carrying out the matrix multiplications seen in (21).

In a typical run it takes 4.5 hours to predict using RR on a machine with 16 cores at 2.60 GHz per core with 64 GB RAM. The RSR predictions are generated alongside at virtually no costs in terms of CPU and memory. The computing time includes computation of the GRM, *P* ^−1^ **X** **X** ^*⊤*^, when *N* ≤ *P* and *P* ^−1^ **X** ^*⊤*^ **X** when *N* > *P*. Given *N* and *P*, failure to exploit (i) the constancy of the GRM and of *P* ^−1^ **X** ^*⊤*^ **X** over the 20 × 37 different combinations of *h* ^2^ and *f*_*C*_ and (ii) the properties of the eigendecomposition which enable the joint evaluation of the 151 values of *λ* we consider dramatically increases the CPU time of RR. In fact, we infer that the less efficient approach yields a CPU time that is at most a factor 20 × 37 × 151 = 111,740 larger than the 4.5 hours we attain (i.e., about 57 years per run). Even worse, when the naive RR approach is applied and also when *P* ≫ *N*, RR predictions cannot be obtained for datasets with more than 50,000 SNPs on the machine we use. Thus, using the efficient approach based on the GRM when *N* ≤ *P* and based on *P* ^−1^ **X** ^*⊤*^ **X** when *N* > *P*, combined with the smart use of eigendecompositions and constancy of GRMs over different combinations of *f*_*C*_ and *h* ^2^ we are able to reduce CPU times from several decades to several hours.

In each run, for each combination of levels we compute the *R* ^2^ of the RSR prediction with the outcome and the *R* ^2^ of the RR prediction with the outcome. *R* ^2^ is measured by the squared sample correlation coefficient between the polygenic score and the outcome in the test set. Our aim is to assess predictive accuracy of RSR and see whether it differs significantly from zero for a wide range of configurations. Moreover, we want to test whether RR provides a significant improvement compared to RSR. Therefore, the performance of RR is measured relative to RSR. That is, we take the log-ratio of the two, given by log⁡⁡(*R*_RR_^2^/*R*_RSR_^2^). This measure is continuously distributed over (−*∞*, +*∞*).

We measure the absolute performance of RSR by the logit transformation of *R*_RSR_^2^/*h*_SNP_^2^; that is,

(33) $$\begin{array}{c} \text{logit}\left(\frac{R_{\text{RSR}}^{2}}{h_{\text{SN}}^{2}}\right)=\log\left(\frac{R_{\text{RSR}}^{2}/h_{\text{SNP}}^{2}}{1-R_{\text{RSR}}^{2}/h_{\text{SNP}}^{2}}\right). \end{array}$$

This measure is also distributed over (−*∞*, +*∞*). The reason for dividing *R*_RSR_^2^ by *h*_SNP_^2^ is that we want to know what part of the genetic variation the polygenic score captures. If *h*_SNP_^2^ is low, for instance, 5%, we consider a polygenic score that attains an *R* ^2^ of 4% to be more impressive than a risk score that explains 10% of the variation in a highly heritable trait (e.g., *h*_SNP_^2^ = 50%). Note that we exclude observations with *R*_RSR_^2^ > *h*_SNP_^2^ as these are uninformative outliers; a polygenic score that “explains” more genetic variation than there actually is is simply wrong.

Regarding the RR penalty, let *R*_RR_^2^(*λ*, *r*) denote the accuracy of RR in run *r*, given penalty *λ*, conditional on some *N*, *P*, *f*_*C*_, and *h* ^2^. Now, let

(34) $$\begin{array}{c} R_{\text{RR},\text{med}}^{2}\left(\lambda \right)=\text{median}\left({\left\{R_{\text{RR}}^{2}(\lambda ,r)\right\}}_{r=1,\ldots ,R}\right) \end{array}$$

denote the median of the RR performance over the runs for a specific value of *λ*. Now, for this combination of *N*, *P*, *f*_*C*_, and *h* ^2^ we take

(35) $$\begin{array}{c} \hat{\lambda }=\underset{\lambda \in \left\{\lambda_{1},\ldots ,\lambda_{L}\right\}}{\text{arg}\;\max}R_{\text{RR},\text{med}}^{2}\left(\lambda \right). \end{array}$$

Thus, for a given combination of levels of factors *λ* is tuned by setting it such that it maximizes the median *R* ^2^ of RR over the runs for the given combination of levels. Based on this procedure, the optimal *R* ^2^ of RR in run *r* is given $R_{\text{RR}}^{2}(\hat{\lambda },r)$. This yields a single measure of accuracy of RR per replication and per combination of levels. This procedure results in a value of *λ* that performs well in 21 independent samples. Hence, it is similar to a value that would result from CV; there is little scope for overfitting. Moreover, since the median is less sensitive to outliers than, for instance, the mean, we make our measure more robust by taking the median over the runs. The reason that we choose for this approach instead of CV is to reduce the computational complexity of the simulation procedure at the expense of having a slightly less elegant approach.

### 9.1. Simulation Results

Table 2 shows the summary statistics of the measure log⁡⁡(*R*_RR_^2^/*R*_RSR_^2^) and of *R*_RSR_^2^. As can be seen, overall the combinations of levels and runs RR seems to outperform RSR on average by about 6%. However, there is much variation in the log-ratio. The lowest log-ratio is −22.2 and the highest is +20.7. Since this ratio is on a log scale this implies a tremendous difference in *R* ^2^. The reason for this is that when either the nominator or the denominator of *R*_RR_^2^/*R*_RSR_^2^ gets close to zero, the log-ratio can attain a large value (in absolute terms). For this reason we excluded log-ratios outside the interval (−1, +1). This leads to a drop in the variance from about 0.4 to 0.04, only at the expense of losing 3.9% of the observed combinations of levels and runs. Moreover, the mean log-ratio hardly changes by removing the outliers. This reduction in variance allows us to display the results in a more insightful manner and ensures further inferences on the relation between our factors (e.g., sample size) and predictive accuracy are not influenced by aberrant observations. For *R*_RSR_^2^ we see that the average *R* ^2^ of about 17% is significantly greater than zero.

**Figure 3:**
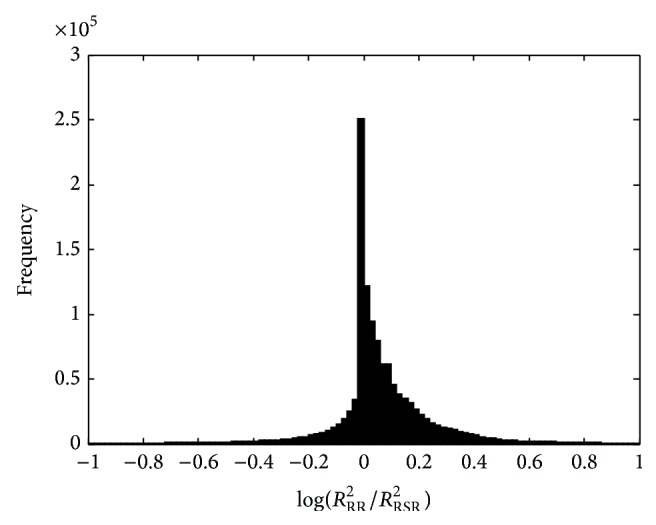
Histogram of log⁡⁡(*R*_RR_^2^/*R*_RSR_^2^) for 21 runs of simulated data, for different values of *N*, *P*, *f*_*C*_, and *h* ^2^. Ridge parameter *λ* is chosen to maximize median *R*_RR_^2^. Values outside (−1, +1) are excluded.

**Figure 4:**
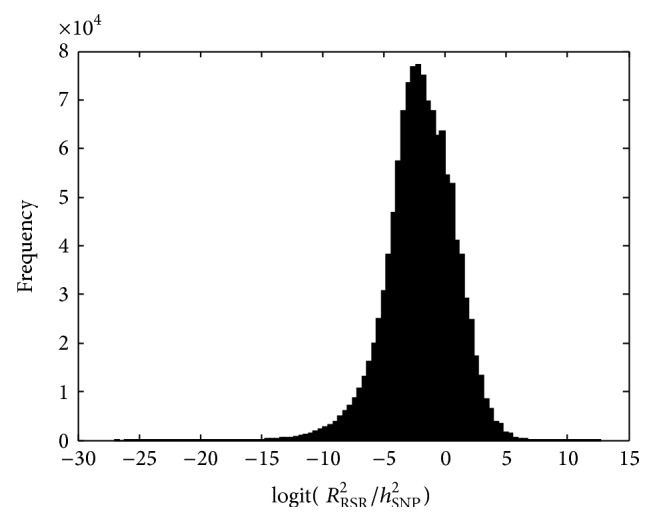
Histogram of logit(*R*_RSR_^2^/*h*_SNP_^2^) for 21 runs of simulated data, for different values of *N*, *P*, *f*_*C*_, and *h* ^2^, excluding observations for which *R*_RSR_^2^ ≥ *h* ^2^.

Figure 3 shows the histogram of log⁡⁡(*R*_RR_^2^/*R*_RSR_^2^) over the combinations of runs and levels inside the range (−1, +1). This histogram confirms that there are long and thin tails. Most mass centers around zero. However, the empirical distribution is slightly skewed to the right, giving rise to the positive average log-ratio. The figure shows that RR often performs better than RSR. Given the fact that RR lies between RSR and OLS, this is not surprising. Using the penalty parameter *λ*, RR tries to find the optimum between these two extremes.  Figure 4 shows the histogram of logit(*R*_RR_^2^/*h*_SNP_^2^) excluding observations for which *R*_RR_^2^ > *h*_SNP_^2^. The observations are smoothly distributed. A value of zero corresponds to an *R* ^2^ equal to half the heritability. Thus, in a substantial proportion of the cases RSR captures more than half of the genetic variation.

**Figure 5:**
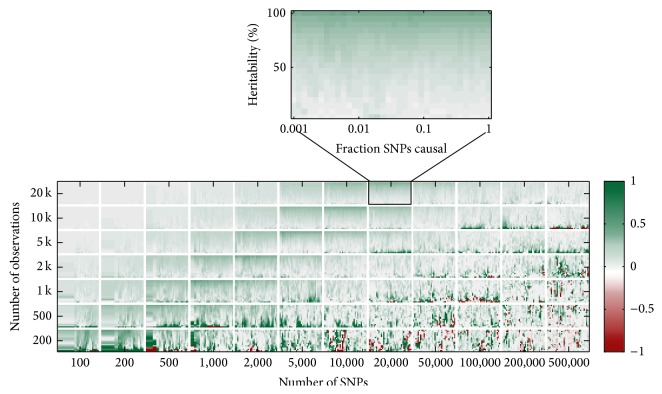
The logarithm of the ratio of the median of the *R* ^2^ over 21 simulations, attained by ridge regression and the classical GWAS approach of RSR, for different combinations of training sample size (*y*-axis), number of SNPs (*x*-axis), trait heritability (*y*-axis in the sub-plots), and fraction of SNPs with a causal effect (*x*-axis in the subplots). Ridge parameter *λ* is chosen to maximize the median *R*_RR_^2^. Values truncated to lie between minus one (red) and plus one (green).

Figure 5 shows the log-ratio of the median *R* ^2^ of ridge regression and of RSR, with values outside the interval (−1, +1) truncated to corresponding extremes of this interval. This truncation is necessary in order for the figure not to be dominated by the outliers. For *N* ≪ *P* (see the lower right block in Figure 5), the performance of RR and RSR is volatile. Sometimes, RR strongly outperforms RSR and sometimes it is the other way round. However, on average RR seems to outperform RSR. As *N* approaches *P* (see the lower left and upper right blocks in Figure 5) RR starts to outperform RSR. There are large regions, where the log of the gain in accuracy is consistently between zero and a half. This corresponds to a relative increase between zero and 65%. For example, for *N* = *P* = 20,000, *h*_SNP_^2^ = 50%, and 200 causal SNPs RSR attains a median *R* ^2^ of 17% and RR 20%, constituting a relative increase of 16%. This gain in accuracy peaks when *N* ≈ *P*.

When *N* ≫ *P* (see the upper left block in Figure 5), the gain in accuracy drops to zero. However, it is unlikely that this pattern, where the gain of RR dies out as *N* keeps increasing, replicates empirically. The reason for this is that the pattern is probably an artefact of the design of the simulation; all SNPs are simulated independent of each other. Even though empirical correlations between SNPs can arise in the simulations, asymptotically there is none. Thus, for sufficiently large *N* (compared to *P*) the standardized simulated SNP data are such that **X** ^*⊤*^ **X** approaches the identity matrix and RR becomes equivalent to RSR (see Section 3). Therefore, the accuracy of RR and RSR does not differ for such extremely large values of *N*. How the performance differs in these large samples when there is linkage disequilibrium in the data remains to be seen.

Table 3 shows the median of the *R* ^2^ of RSR and that of RR relative to RSR for combinations of sample size and the number of genotyped SNPs that are typically seen in a GWAS (e.g., *N* = 10,000, *P* = 500,000). We see that for these data dimensions a trait with a heritability of 50% has a classical polygenic score which on average only explains 0.5% of the total phenotypic variation. Moreover, RR yields a relative increase of just 2.9%. This increase gives an absolute *R* ^2^ of 0.51% for RR. This observation clearly illustrates that the so-called missing heritability [45] is hard to find, even under a very simple data generating process, that is, a process for which we are sure that both RSR and RR should asymptotically capture all genetic variation.

### 9.2. Modelling the Simulation Results

To understand the relation between the various factors in the simulation study and the gain in predictive accuracy by RR we fit a linear model to the logarithm of the ratio *R*_RR_^2^/*R*_RSR_^2^ for all replications and for all considered levels of factors, such as sample size. Moreover, in order to obtain the *R* ^2^ of RSR as a benchmark we also fit a linear model, the logit transformation of *R*_RSR_^2^.

The results in the previous section indicate that the relation between sample size *N* and the performance is nonlinear. The relation seems to exhibit an inverted U-shape. For this purpose, we include log⁡(*N*) and its square as regressors. Moreover, the location of the peak depends on the number of SNPs, implying that the parameters of regressors related to sample size depend on *P*. Consequently, interactions between *P* and *N* are added to the model. By symmetry of Figure 5, similar arguments hold for the performance as function of *P*. Based on this argument we consider up to three-way interactions between the regressors.

In addition, we see in many subplots of Figure 5 that the gain in predictive accuracy differs systematically between low, intermediate, and high heritabilities. Therefore, heritability is included as regressor. Finally, although the effect of the fraction of causal SNPs is hard to judge from Figure 5, we include this factor as regressor as well.

Both outcomes are modelled as a linear function of the aforementioned basic regressors. These regressors are reported in Table 4. We consider models ranging from merely an intercept up to all 3-way interactions between the explanatory variables. We choose the model that minimizes the Bayes information criterion (BIC) [59].

Table 5 reports the BIC values of the respective models. On the basis of these values we find that a model including all three-way interactions is most appropriate, both in case of the log-ratio and in case of the logit of the performance of RSR relative to the heritability. The model for the gain in accuracy of RR relative to RSR can explain approximately 12% of the variation in this measure on the basis of sample size and the other regressors. The model for the accuracy of RSR can explain about 61%.

A likely reason for the fact that we can explain far more variation in the *R* ^2^ of RSR than in the gain of RR relative to RSR is the following. In case both the *R* ^2^ of RR and RSR are to a large extent influenced by our factors in a similar way, taking the log-ratio basically eliminates these common effects. What then remains is a measure over which the factors have less predictive power than over the absolute *R* ^2^ measure.

**Figure 6:**
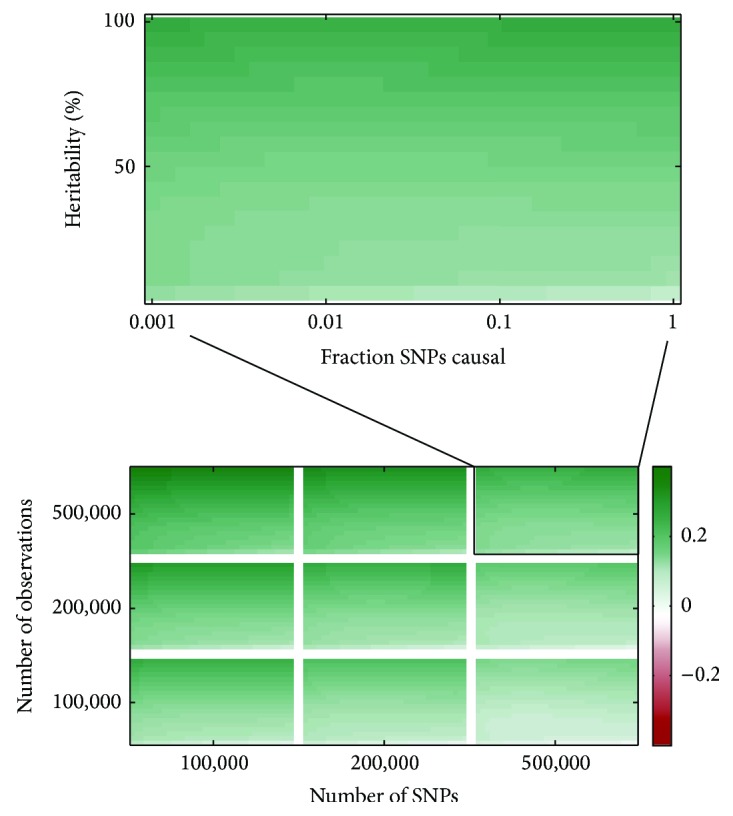
The predicted logarithm of the ratio of the *R* ^2^ attained by ridge regression and the classical GWAS approach of RSR, based on a model fitted to this measure in 21 runs of simulations. Predictions are shown for different combinations of training sample size (*y*-axis), number of SNPs (*x*-axis), trait heritability (*y*-axis in the subplots), and fraction of SNPs with a causal effect (*x*-axis in the subplots). Predicted values are not truncated. Deep green represents the highest prediction.

Using the parameters estimates of the models we predict the log-ratio of *R*_RR_^2^ and *R*_RSR_^2^ as well as *R*_RSR_^2^ for sample sizes between 100,000 and 500,000 individuals and the number of SNPs between 100,000 and 500,000. For heritability and the fraction of causal SNPs we use the ranges considered in the initial simulations. The resulting predictions of the gain in accuracy are displayed in the heatmap in Figure 6.

In addition, point estimates of *R*_RR_^2^/*R*_RSR_^2^ and *R*_RSR_^2^ are reported together with confidence intervals in Table 6. There are three groups of predictions. In the first group *P* = 500,000, *h* ^2^ = 50%, and *N* varies from 100,000 to 500,000. In the second group *N* = 500,000 and *P* varies from 100,000 to 500,000. In the last group *P* = *N* = 500,000 and *h* ^2^ ranges from 25 to 75%.

Results from Figure 6 and Table 6 indicate that in most cases RR is expected to yield a relative increase in *R* ^2^ between 10% and 20% for sample sizes ranging between 100,000 and 500,000 individuals. All increases in accuracy are greater than zero at a 5% significance level. Moreover, RSR attains values of *R* ^2^ ranging between 15% and 75%. As an example, in case of 200,000 individuals and 500,000 SNPs, for a trait with *h*_SNP_^2^ = 50% the *R* ^2^ of RSR is expected to be 33.7% and the *R* ^2^ of RR 37.3%.

Regarding these findings, combining the *R* ^2^ attained by RSR with the relative increase by RR yields expected values of the *R* ^2^ of RR which in some cases surpass *h* ^2^. In practice this cannot be true. In case a trait has an *h* ^2^ of 50% it is not possible to consistently predict more than 50% of the phenotypic variation on the basis of SNP data. This seems to indicate that our estimates are somewhat optimistic. Nevertheless, for the ranges in which we actually simulated data (i.e., *N* ≤ 20,000 and *P* ≤ 500,000) RSR is able to attain a substantial *R* ^2^ when *N* ≈ *P* and RR is able to considerably increase the *R* ^2^. For instance, at *h* ^2^ = 50% and *N* = *P* = 20,000, with 200 causal SNPs the median *R* ^2^ of RSR is 17%, and the median *R* ^2^ of RR is 20%. This constitutes a relative increase in *R* ^2^ of about 16%. As shown in Figure 5, this pattern seems to persist while *N* ≈ *P*. Hence, at the very least, the expectation that RR improves the *R* ^2^ of RSR considerably for large samples (e.g., *N* ≈ *P* ≈ 500,000) is not unreasonable.

## 10. Conclusions and Discussion

Ridge regression is a flexible technique that can be used to estimate the association between a set of *P* SNPs and an outcome observed for *N* individuals, even when *P* ≫ *N*. When the ridge penalty is equal to the ratio of the noise variance and the variance of random SNP effects in a mixed linear model, prediction using the weights from ridge regression is equivalent to the best linear unbiased prediction used in animal breeding, agricultural science, and more recently also human genetics.

Ridge regression can be perceived as a method that partially accounts for linkage disequilibrium between markers. For a sufficiently low penalty the method fully accounts for linkage disequilibrium and is therefore equivalent to the OLS estimator of the multiple regression problem using all SNPs jointly. On the other hand, for a sufficiently high penalty, in terms of predictions ridge regression ignores linkage disequilibrium and is therefore equivalent to the approach of a simple regression per SNP, which is common in a GWAS.

Using standard results from, for instance, machine learning and animal breeding, prediction using ridge regression can be shown to constitute solving an equation with *N* unknown weights and applying these weights to a measure of relatedness of individuals out of sample and in sample. Formulating ridge regression this way makes it a computationally efficient technique, even for a large number of SNPs.

As with multiple regression and GWAS predictions, ridge regression can account for the presence of confounding variables, such as age, gender, and population structure. Moreover, such corrections can again be implemented at low computational costs.

When the shrinkage parameter is unknown ridge prediction can be formulated such that predictions for different values of this parameter can be generated in a single step, requiring the eigendecomposition of an *N* × *N* matrix only once. This expression allows the researcher to efficiently carry out procedures, such as cross-validation, to tune this parameter.

Finally, ridge regression prediction is amenable to a wide array of advanced techniques. First, using the kernel trick from machine learning, nonlinear effects such as dominance and epistasis can easily be incorporated in the prediction model. Moreover, in a Bayesian spirit, results from earlier studies can be used to give a prior weight to SNPs in the ridge regression prediction. Similarly, when prior information is not available, in-sample information can be used to discount SNPs differently, yielding a heteroskedastic ridge regression prediction.

Empirical findings so far seem to suggest that for current sample sizes the performance of plain vanilla ridge regression is very similar to that of the repeated simple regression approach used in a GWAS. This raises two questions. First, how do more advanced ridge regression approaches perform? Second, how will the plain version of ridge regression perform in upcoming large scale initiatives, such as biobanks?

Using a suite of simulations we consider the second question. We confirm the finding that for most current studies, with sample sizes usually below 10,000 individuals and more than 500,000 SNPs, ridge regression hardly outperforms the classical GWAS approach. For a sample of 10,000 observations, with 500,000 SNPs of which 5,000 causal, for a trait with a heritability of 50%, the median *R* ^2^ in 21 independently simulated datasets is 0.5% for repeated simple regression and 0.51% for ridge regression. This resonates with the finding that the main determinant of predictive accuracy of the polygenic score is the sample size of the training set (e.g., [60, 61]). As long as *N* ≪ *P*, there seems to be little advantage of advanced approaches, such as ridge regression, over the classical GWAS approach [61].

However, by analyzing the difference in accuracy of the classical approach and ridge regression for different values of *N*, *P*, trait heritability, and the fraction of causal variants, we are able to extrapolate the performance of ridge regression for large scale initiatives. For a sample size of 200,000 individuals and 500,000 SNPs, we find that in a trait with 50% heritability and with 5,000 causal variants the polygenic score of a GWAS is expected to explain 34% of the phenotypic variation, whereas ridge regression is expected to capture about 37%. Thus, in this scenario ridge regression is expected to capture about 75% of the genetic variation, whereas the classical approach captures 67%.

However, these predictions are rather coarse. They depend highly on the model being fitted (e.g., by including interactions between the number of individuals, SNPs, and heritability). This observation comes as no surprise; we extrapolate quite a bit outside the interior of the levels of the factors that were considered in the simulations (e.g., *N* ≤ 20,000). However, one thing that remains unchanged even under different specifications of the models that try to explain the accuracy of respective methods is that ridge regression outperforms the repeated simple regression approach in all large scale samples considered.

A final note is concerned with the independence of the loci. In the present simulations at most 500,000 truly independent markers were used. As a result, all carry their own idiosyncratic bit of information about the genetic relationship of individuals in the data. As is shown, however, by Yang et al. [62], in real data with linkage disequilibrium taking a random subset of 60% or more of the SNPs from a grand set of 295 k SNPs yields heritability estimates of human height highly similar to estimates based on the full set; apparently adding more markers hardly changes the genetic relatedness estimates.

The findings of Yang et al. [62] illustrate that there might be a limited number of SNPs that can make a meaningful contribution to the SNP-based measure of genetic relationship. After this “effective number of SNPs” [63], new SNPs are primarily repeating the story that has been told by previous SNPs already. Therefore, even with many millions of SNPs (e.g., in imputed data), the resulting genetic relatedness estimates are highly similar to those obtained from a considerably smaller set of SNPs. Consequently, if this “effective number of SNPs” exists this implies that for large scale initiatives the performance of ridge regression relative to repeated simple regression might be similar to what we have observed in our simulations when *N* ≈ *P*, even when in fact *P* is far greater still than *N*. Such a proposition would need to be tested either in empirical work or by means of simulations using actual genotype data in which linkage disequilibrium is present.

The use of GWAS data for the prediction of complex traits based on sample sizes far below 100,000 individuals yields genetic risk scores with little predictive accuracy, regardless of whether one applies the classical GWAS approach or ridge regression. However, as sample sizes approach the “effective number of SNPs” we expect the polygenic risk score based on repeated simple regression to be able to explain a substantial proportion of the normal genetic variation. Moreover, under this scenario prediction using ridge regression is likely to outperform the classical GWAS predictions significantly. Bearing in mind that ridge regression is amenable to include nonadditive genetic variance in the prediction model it is therefore not unlikely that ridge regression will make an even more substantial contribution to the accuracy of polygenic scores in traits where epistasis and dominance are expected to play an important role.

## Figures and Tables

**Algorithm 1:**
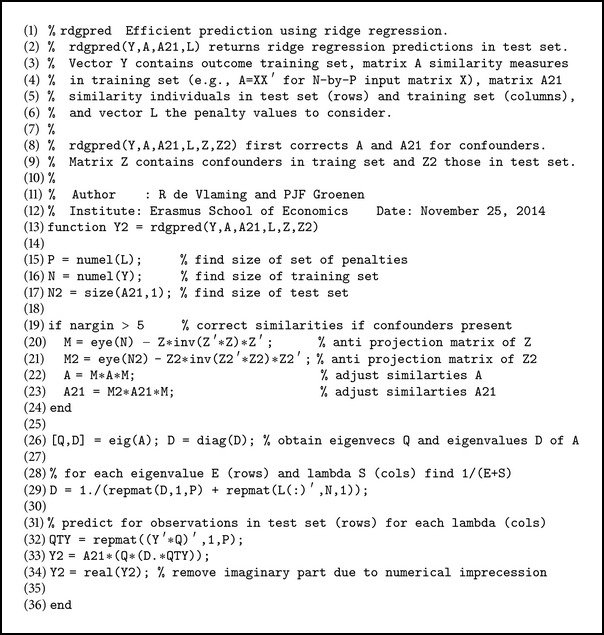
MATLAB code for efficient ridge regression prediction: rdgpred.m.

**Table 1:** Factors and levels of the simulation study. *h*_SNP_^2^: heritability simulated phenotype, *N*: sample size training set, *P*: number of SNPs, and *f*_*C*_: fraction of SNPs causal.

Factors	Levels	Number of levels
*N*	{200; 500; 1,000; …; 10,000; 20,000}	7
*P*	{100; 200; 500; …; 100,000; 200,000; 500,000}	12
*f*_*C*_ (%)	{0.1; …, 100}	37
	(Linear increases on logarithmic scale)	
*h*_SNP_^2^ (%)	{5; 10; 15; …; 100}	20

**Table 2:** Summary statistics of log⁡(*R*_RR_^2^/*R*_RSR_^2^) for the full set of observed log-ratios and for the subset excluding log-ratios outside (−1, +1), and *R*_RSR_^2^ for the full set and for the subset excluding observations for which *R*_RSR_^2^ ≥ *h*_SNP_^2^. Results stem from all combinations of the levels of the factors in the simulation design, with 21 replications per combination.

Outcome	Restriction	Count	(% total)	Mean	Var.	Min	Max
log⁡(*R*_RR_^2^/*R*_RSR_^2^)	None	1,305,360	(100.0%)	0.065	0.403	−22.2	20.7
log⁡(*R*_RR_^2^/*R*_RSR_^2^)	∈(−1, +1)	1,254,168	(96.1%)	0.060	0.041	−1.00	1.00

*R*_RSR_^2^	None	1,305,360	(100.0%)	0.177	0.058	0.000	0.997
*R*_RSR_^2^	<*h*_SNP_^2^	1,239,721	(95.0%)	0.160	0.051	0.000	0.997

**Table 3:** The observed median of the *R* ^2^ of RSR and of RR relative to RSR, over 21 simulations, for different sample sizes (*N*), number of SNPs (*P*) of which 1% causal, and heritabilities (*h*_SNP_^2^). Ridge parameter *λ* is chosen to maximize the median *R*_RR_^2^.

*N*	*P*	*h*_SNP_^2^	Median *R*_RSR_^2^	Median *R*_RR_^2^/ median *R*_RSR_^2^
5 k	500 k	0.50	0.003	1.078
10 k	500 k	0.50	0.005	1.029
20 k	500 k	0.50	0.009	1.038

10 k	100 k	0.50	0.027	1.079
10 k	200 k	0.50	0.011	1.000
10 k	500 k	0.50	0.005	1.029

10 k	500 k	0.25	0.001	1.000
10 k	500 k	0.50	0.005	1.029
10 k	500 k	0.75	0.011	1.011

**Table 4:** Regressors used to explain the predictive accuracy of RR and RSR.

Regressor	Captures
Intercept	Level
log⁡(*N*)	Effect sample size
log⁡(*P*)	Effect number of SNPs
log⁡(*C*)	Effect of number of causal SNPs (*C*)
log⁡(*f*_*C*_)	Effect of fraction of SNPs causal
log⁡(h^2^)	Effect of heritability

**Table 5:** Bayes information criterion (BIC) and the proportion of explained variance (*R*_model_^2^) of the model for the gain in predictive accuracy of RR relative to RSR and the model for the performance of RSR, over different combinations of levels of the factors sample size, number of SNPs, fraction of causal SNPs, and heritability. Lowest BIC printed bold.

Outcome	Regressors (number of regressors)	Number of observations	*R*_model_^2^	BIC
log⁡(*R*_RR_^2^/*R*_RSR_^2^)	Intercept (1)	1,254,168	0.0%	−3.998 · 10^6^
log⁡(*R*_RR_^2^/*R*_RSR_^2^)	Regressors in Table 4 (5)	1,254,168	4.7%	−4.058 · 10^6^
log⁡(*R*_RR_^2^/*R*_RSR_^2^)	& 2-way interactions (15)	1,254,168	8.4%	−4.107 · 10^6^
log⁡(*R*_RR_^2^/*R*_RSR_^2^)	& 3-way interactions (35)	1,254,168	12.4%	−4.163 · 10^6^

logit⁡(*R*_RSR_^2^/*h*_SNP_^2^)	Intercept (1)	1,239,721	0.0%	2.542 · 10^6^
logit⁡(*R*_RSR_^2^/*h*_SNP_^2^)	Regressors in Table 4 (5)	1,239,721	48.6%	1.717 · 10^6^
logit⁡(*R*_RSR_^2^/*h*_SNP_^2^)	& 2-way interactions (15)	1,239,721	56.3%	1.515 · 10^6^
logit⁡(*R*_RSR_^2^/*h*_SNP_^2^)	& 3-way interactions (35)	1,239,721	60.9%	1.379 · 10^6^

**Table 6:** Predictions of the predictive accuracy of RSR and of the gain in predictive accuracy of RR compared to RSR in large scale samples (e.g., *N* ≥ 100,000) based on a linear model with sample size, number of SNPs, fraction of causal SNPs, and heritability as predictors. 95% confidence intervals (CI) are reported in parentheses, with the middle value indicating the point estimate. 1% of the SNPs are assumed to be causal.

*N*	*P*	*h*_SNP_^2^	95% CI *R*_RSR_^2^	95% CI *R*_RR_^2^/*R*_RSR_^2^
100 k	500 k	0.50	(0.139; 0.146; 0.153)	(1.062; 1.070; 1.079)
200 k	500 k	0.50	(0.324; 0.337; 0.349)	(1.094; 1.107; 1.121)
500 k	500 k	0.50	(0.473; 0.478; 0.482)	(1.142; 1.167; 1.193)

500 k	100 k	0.50	(0.486; 0.488; 0.490)	(1.218; 1.244; 1.270)
500 k	200 k	0.50	(0.482; 0.485; 0.488)	(1.193; 1.218; 1.244)
500 k	500 k	0.50	(0.473; 0.478; 0.482)	(1.142; 1.167; 1.193)

500 k	500 k	0.25	(0.205; 0.212; 0.218)	(1.110; 1.135; 1.160)
500 k	500 k	0.50	(0.473; 0.478; 0.482)	(1.142; 1.167; 1.193)
500 k	500 k	0.75	(0.733; 0.736; 0.739)	(1.191; 1.218; 1.245)
